# A *de novo* mutation in *RAB11A* is associated with neurodevelopmental disorder accompanied by variable multisystem abnormalities

**DOI:** 10.3389/fgene.2025.1636206

**Published:** 2025-09-01

**Authors:** Huiting Zhang, Jingtao Zhang, Xue Ma, Zhehui Chen, Ying Jin, Mengqiu Li, Hui Dong, Feng Gu, Yao Zhang, Yanling Yang

**Affiliations:** ^1^ Children’s Medical Center, Peking University First Hospital, Beijing, China; ^2^ Department of Pediatrics, Women and Children’s Hospital, School of Medicine, Xiamen University, Xiamen, China; ^3^ Key Laboratory of Model Animals and Stem Cell Biology in Hunan Province, Engineering Research Center of Reproduction and Translational Medicine of Hunan Province, Medical Center, Hunan Normal University, Changsha, China

**Keywords:** rab11a, neurodevelopmental disorder, zebrafish, motor deficits, crispant

## Abstract

**Introduction:**

RAB11A, a Rab GTPase, is crucial for intracellular transport and recycling. Recently, *RAB11A* mutations have been found to be associated with neurodevelopmental disorders in cohorts. At present, there are still no effective treatment methods for NDDs caused by *RAB11A* deficiency, thus, identifying pathogenic mutations and generating disease models is crucial for advancing our understanding of these conditions.

**Methods:**

We analyzed the clinical presentation of a 4-year and 4-month-old boy with a *de novo RAB11A* mutation c.370A>G. To examine the consequences of RAB11A mutation during early embryonic development, we disrupted the homologous rab11a gene using CRISPR/Cas9 in zebrafish.

**Results:**

The affected boy who exhibited intellectual disability showed phenotypic features including cerebral atrophy, obesity, motor disability and abnormal muscle tone. Protein structure predictions indicated that *RAB11A* mutation affected protein stability and enzymatic activity. CRISPR/Cas9-mediated *rab11a* deficiency in zebrafish larvae significantly reduced brain, forebrain, and midbrain size.

**Conclusion:**

Our study collectively demonstrated that the *RAB11A* mutation c.370A>G is associated with neurodevelopmental disorders, characterized by motor deficits and brain anomalies. Additionally, we have successfully developed a zebrafish model to recapitulate these neurodevelopmental disorders associated with *RAB11A* deficiency, offering a valuable genetic resource for further investigation into this disease.

## 1 Introduction

Children with neurodevelopmental disorders (NDDs) often experienced challenges in language, speech, motor skills, behavior, memory, learning, and other neurological functions. NDDs affect nearly 2% of the global population, impacting individuals throughout their lives (Moreau et al., 2023). Identifying the underlying causes of NDDs is crucial for advancing treatment, with genetic mutations playing a major role (Kiser et al., 2015). Many gene mutations such as *ARID1B, SATB2, SYNGAP1, ANKRD11, SCN1A, DYRK1A STXBP1, MED13L*, were widely found to be associated with NDDs, and novel genes like *RNU4-2* were emerging (Deciphering Developmental Disorders Study, 2015; Coe et al., 2019; Maia et al., 2021; Chen et al., 2024). *RAB11A* has been reported as a pathogenic gene in large-scale cohorts of individuals with intellectual disability and developmental delay by Hamdan et al., in 2017, although its molecular mechanisms remain underexplored due to the lack of large cohorts and animal models (Deciphering Developmental Disorders Study, 2015; Hamdan et al., 2017). *RAB11A* was more recently studied in 2024 by Borroto and Hamdan, who reported a cohort of 16 individuals affected by *RAB11A* mutations (Borroto et al., 2024). These patients characterized by NDD accompanied by variable multisystem abornmalities including motor deficits, obesity, muscle tonus abnormalities, brain structural anomalies, cardiac defects and vision impairments.

The human RAB11A gene, located on chromosome 15, encodes a small GTPase protein consisting of 216 amino acids (Sultana and Novotny, 2022). RAB11A protein primarily localizes to the cell membrane, Golgi apparatus, endoplasmic reticulum, endosomes, and dendritic spines (Jain et al., 2023). As a Rab GTPase, RAB11A is a key regulator of intracellular membrane transport and recycling, participating in protein complex formation as well as endocytosis and exocytosis. It functions as a cellular switch based on its GDP/GTP binding state (Pasqualato et al., 2004). It co-localizes with recycling endosomes, markers such as HSP90 and RAB39B (Tran et al., 2022). Endocytosis and vesicle transport are fundamental processes that regulate development, disease, and cellular homeostasis. The endosome–lysosome transport pathway is pivotal in maintaining normal neurodevelopment (Ascano et al., 2012). While the neuronal functions of *RAB11A* remain to be fully understood, emerging evidence suggests its involvement in various neuronal processes (Blackman et al., 2020; Venugopal et al., 2020). Downregulation of *RAB11A* expression has been observed to reduce the expression of neuronal differentiation markers and to inhibit the elongation of neurite-like processes in both N1E-115 cells and primary cortical neurons (Fukatsu et al., 2024). Puri et al. described the mechanism by which WIPI2, through its binding to RAB11A, localizes to the autophagic precursor membrane. The loss of RAB11A impairs the recruitment and assembly of the autophagy machinery (Puri et al., 2018). Global ablation of the mouse Rab11a gene impairs early embryogenesis, with *Rab11a*-null embryos dying *in utero* at the implantation stage (Yu et al., 2014a; Yu et al., 2014b). Mice with intestines-specific or inner ear-specific knockout (KO) of *Rab11a* exhibit postnatal lethality or abnormal development, respectively (Sobajima et al., 2014; Chen et al., 2021). *Rab11a* Morpholino anti-sense oligos zebrafish embryos show altered body curvature (Clark et al., 2011). And double knockout of *rab11a* and *rab11ab* zebrafish showed abnormal lens development in 36hpf (Hao et al., 2020). However, there was few animal models to study the *RAB11A*-NDD (*RAB11A* related neurodevelopmental disorders), further studies to observe phenotypes and molecular basis in animal models are required.

Herein we reported a case involving a *de novo RAB11A* mutation. Additionally, we established a *rablla* deficient zebrafish model. This model exhibits various morphological and neurobehavioral abnormalities, including decreased central nervous system (CNS) area, hyperactive spontaneous swimming and spontaneous electrical discharges. Our findings provide new insights into *RAB11A*-related neurodevelopmental disorders.

## 2 Materials and methods

### 2.1 Patient

**FIGURE 1:**
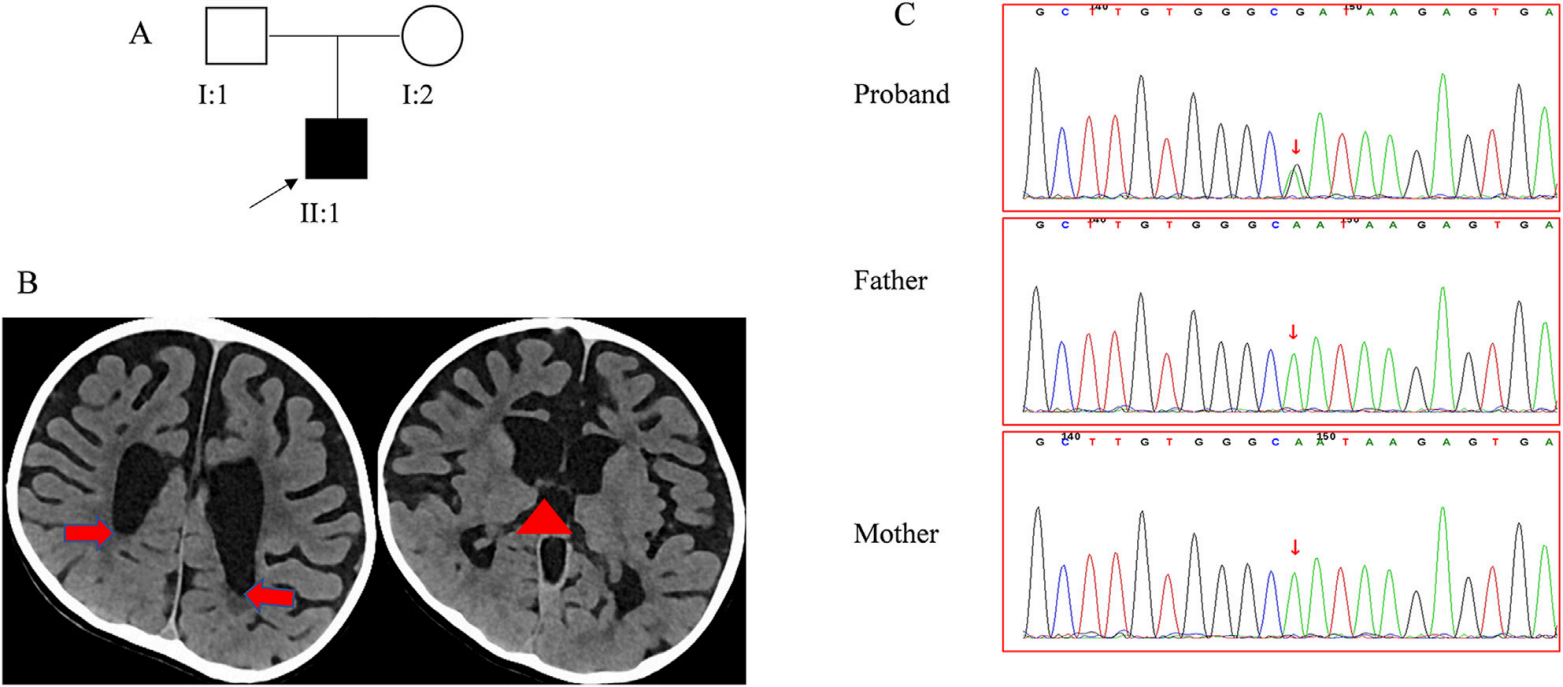
Clinical data for the patient **(A)**. The pedigree of family 1 segregates CM. The arrow points to the proband. **(B)** The CT scan reveals the absence of the corpus callosum. The red arrow points to the dilated posterior horn of the lateral ventricle, and the red triangle indicates the upwardly enlarged third ventricle. **(C)** Sanger sequencing confirmed the *RAB11A* mutation in family members.

A 4-year and 4-month-old boy presenting with global developmental delay was admitted to Our Hospital. He was the firstborn child of non-consanguineous, healthy parents (Figure 1A). To investigate the underlying etiology, a comprehensive set of analyses was performed, including clinical investigations, routine laboratory tests, metabolic studies, brain imaging, and gene sequencing.

### 2.2 Whole genome sequencing

Genomic DNA was extracted using a kit, followed by random shearing with an ultrasonic disruptor and subsequent library construction. High-throughput sequencing was then performed on the Illumina HiSeq X Series platform. Raw data were processed to remove low-quality reads, and BWA software was utilized for read alignment to the human genome reference sequence hg19. Single nucleotide polymorphisms and insertions/deletions were identified using GATK software, with subsequent frequency analysis against a normal population database. Suspicious mutations were then assessed for potential harm using different online prediction software (SIFT, PolyPhen-2, and Mutation Taster). Finally, the pathogenicity of candidate mutations was graded as per the guidelines of the American College of Medical Genetics and Genomics (Altassan et al., 2019). Sanger sequencing was performed to verify these candidate mutations.

### 2.3 Three-dimensional protein structure modeling

Three-dimensional protein structure models were built using SWISS-MODEL. Sequences were obtained from UniProt (P62491, 216 amino acids) for predicting Rab11a amino acid changes. Structural comparison and mutagenesis analysis was performed using PyMOL 2.3.0, and changes in the interactions at the sites pre- and post-mutation were compared. Data related to amino acid conservation were obtained from the Conserved Domain Database (Wang et al., 2023).

### 2.4 Zebrafish maintaince

Adult zebrafish were maintained in tanks with circulating water at 28°C on a 14/10 h light/dark cycle and fed twice a day. Zebrafish embryos were obtained by mating adult fish through standard methods (Westerfield, 2000). Larvae were raised in embryo media consisting of 0.03% Instant Ocean and 0.0002% methylene blue in reverse osmosis-distilled water. Fluorescent imaging experiments were performed on the Tg (HuC:EGFP) line (purchased from China Zebrafish Resource Center, CZRC) (Park et al., 2000). All procedures were performed in accordance with the Guide for the Care and Use of Animals (National Research Council, 2011) and were approved by the Zhejiang Province Experimental Animal Use License (License Number: SYXK(Zhe)2024–0021).

### 2.5 Imaging and phenotyping

Microscopic morphological observation: Tg (HuC-GFP) zebrafish larvae at 5 days post-fertilization (dpf) was individually placed in an 18-well culture plate (1 normally swimming larva per well), ensuring a horizontal position with the back facing upwards. Bright-field photomicroscope was set at ×2 magnification, while fluorescence photomicroscope was set at ×4 magnification. Images were captured using a Nikon SMZ800N stereo microscope equipped with a Touptek high-resolution CCD camera. The interocular distance relative to body length and brain fluorescence area in the images were measured using ImageJ.

The experiment included a Cas9 control group and a *rab11a* crispant group, with both groups simultaneously analyzed in the same 96-well plate.

### 2.6 Electrophysiological testing

Zebrafish larvae at 5-6 dpf were subjected to electrophysiological testing (Griffin et al., 2016). The test was divided into Cas9 control group and rab11a knockdown group. The 300 μM pancuronium bromide was added to the sample plate to narcotize the zebrafish larvae. After 3 min, the zebrafish were fixed on the back side up with 2% low-melting point agarose gel. After the agarose solidified, it was transferred to the experimental recording tank, and culture medium E was dropped to cover the agarose and the reference electrode. Brain field potentials were measured from the optic tectum with a glass microelectrode containing 2 M sodium chloride solution. The electrical signals were recorded with an electrophysiological signal amplifier with a high impedance (A-M Systems). In the experiment, the electrical signals were digitized by an analog-to-digital converter, the sampling frequency was 10 kHz, the filter was set to 1 Hz to 5 kHz, and the measurement time was 15 min. The data were collected and analyzed by DClamp software (https://sites.google.com/site/dclampsoftware/home).

### 2.7 Statistical analysis

Upon completion of all phenotypic experiments, the editing efficiency of the experimental samples was assessed. Samples that failed sequencing or exhibited low editing efficiency (<5%) were excluded from statistical analysis. Statistical analysis and graphing were performed using GraphPad Prism v8. Unless stated otherwise, error bars represent standard deviation, with P < 0.05 indicating statistical significance.

## 3 Results

### 3.1 Clinical data

The patient, born full-term at the gestational age of 41 weeks (Figure 1A) with a birth weight of 4.3 kg, showed no signs of asphyxiation and had a normal Apgar score. His mother’s pregnancy was uncomplicated, and routine prenatal screenings did not reveal any abnormalities. Since infancy, the patient exhibited a strong appetite, with his body mass index ranging from 19.00 to 22.62 kg/m^2^. His height development remained within the normal range (0–1 SD). At birth, his occipitofrontal circumference (OFC) was normal but later fluctuated between −2 to 1 SD. Motor and cognitive delays were apparent early, resulting in the diagnosis of psychomotor retardation at 11 months. Despite starting rehabilitation at 5 months of age, progress was limited. He achieved head control at 11 months and could sit unsupported at 3 years of age. By 3 years and 5 months, he could stand and walk with support, but independent walking had not achieved by 4 years and 4 months.

In his first year, the patient exhibited hypotonia and lower limbs weakness, though tendon reflexes were normal. After 1 year of age, hypertonia emerged, along with hyperreflexia, ankle clonus, and positive Babinski sign, while axonal hypotonia persisted. Treatments with baclofen and L-dopa yielded no significant improvement. The patient did not experience seizures, but electroencephalogram findings were abnormal. Blood tests and evaluations of the cardiovascular, endocrine (including insulin, thyroid and adrenocorticotropic hormone), and skeletal systems revealed no abnormalities. Physical malformations were also absent. The patient underwent two sets of brain imaging studies. At the age of 1 year, his brain MRI revealed a thin corpus callosum, narrow gyri, deep sulci, and dilated bilateral ventricles. At the age of 3 years and 7 months, his brain CT scan showed significant atrophy of the bilateral brain and enlarged third ventricles, while the corpus callosum appeared normal (Figure 1B). With the four-year follow-up, his skills developed very slowly without regression.

### 3.2 *De novo RAB11A* mutations

**FIGURE 2:**
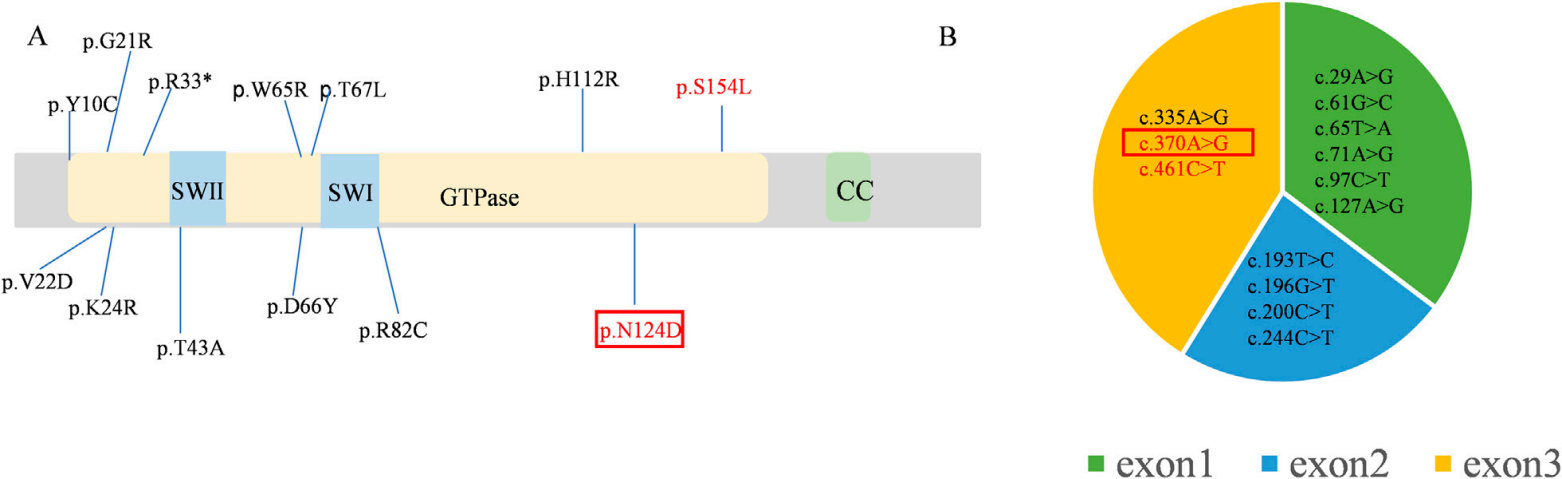
The mutations of the *RAB11A* (NM_004663.5) **(A)** Lollipop graph shows pathogenic mutations in RAB11A protein reported in the literature and identified in this study. **(B)** The pie chart shows the distribution of mutations in the exons. The repeatedly reported mutations are marked in red and the mutation of our proband was pointed in red box.

A *de novo* mutation in the *RAB11A* (NM_004663.5), c.370A>G (p. Asn124Asp), was detected through both whole genome sequencing and Sanger sequencing (Figure 1C). This mutation was absent in reference population databases, including the Genome Aggregation Database (gnomAD), the 1000 Genomes Project, and the Exome Aggregation Consortium (ExAC). Following the criteria set by the American College of Medical Genetics and Genomics, the mutation was classified as likely pathogenic. Several *in silico* predictive tools also indicated that the mutation was damaging. Notably, the same mutation has been found in another girl with NDD (Borroto et al., 2024). Up to now, 13 pathogenic mutations of *RAB11A* were identified from 17 patients (Figure 2A), and 11 mutations were found once. The c.370A>G was found twice, and the c.461C>T was found four times. All these mutations distributed exon 1 to exon 3. No mutations were found in exon 4 and exon 5 (Figure 2B).

### 3.3 Structural modeling and *in silico* analysis of RAB11A protein

**FIGURE 3:**
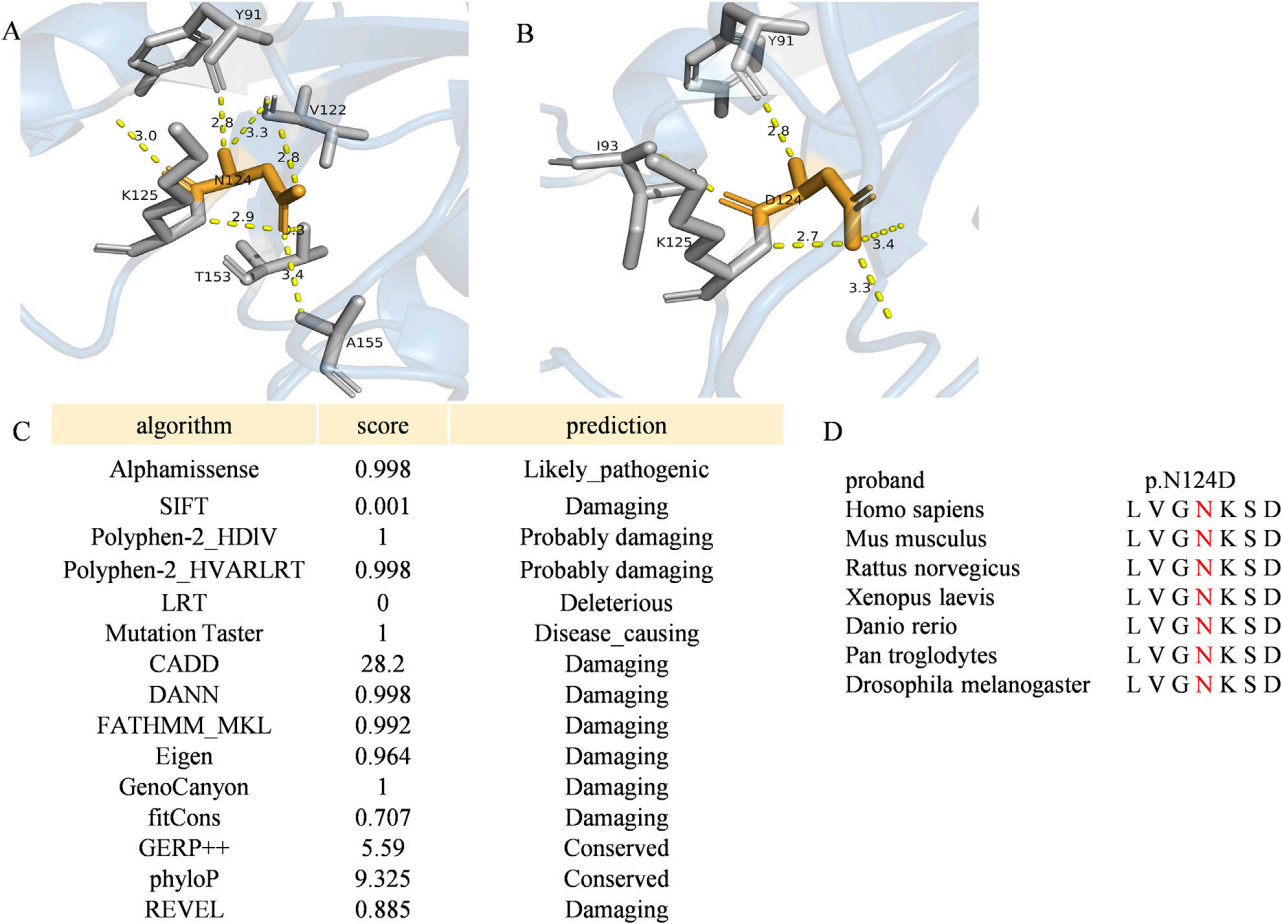
The variant p. Asp124Asn of RAB11A **(A,B)** The angles among the nearest contact residues and the wild-type or mutated residues were also measured. Wild-type and mutated residues are depicted by orange sticks, while contact atoms are represented by gray colors. Yellow dotted lines indicate hydrogen bonds. **(C)** Pathogenicity prediction of the RAB11A missense variant (c.370A>C) using multi-algorithm consensus. Functional impact of the RAB11A mutation (p.N124D) was evaluated by bioinformatic tools based on distinct principles. Evolutionary conservation: GERP++, phyloP. Structural/physicochemical properties: SIFT, PolyPhen-2_HDIV/HVAR, FATHMM-MKL. Machine learning integration: Alphamissense, REVEL, CADD, DANN, Eigen. Pathogenicity probability: MutationTaster, LRT, fitCons, GenoCanyon. **(D)** Multiple-sequence alignment in RAB11A from different species. The variant was pointed out in red. Conserved protein sequence alignment across 7 species validates critical residue position.

Protein structure predictions have highlighted the impact of the c.370A>G mutation on the stability and function of RAB11A protein. Alterations were observed in bond angles, hydrogen bonding patterns, molecular distances, and interactions with other molecules, potentially compromising the protein functionality and its ability to interact with binding partners (Figures 3A,B). RAB11A contains a conserved GTPase domain, and our patient showed a variation at the 124th amino acid, part of the conserved GTP binding site with Mg^2+^ and the G4 box region. This variation affects the number of hydrogen bonds, thereby impacting RAB11A function (Eathiraj et al., 2006; Shin et al., 2016). And the N124 is located in the GTP binding domain of RAB11A, and its charge change (wild-type asparagine: Asn, polar uncharged to aspartic acid: Asp, acidic charged) may interfere with GTP hydrolysis or the binding of effector proteins. The prediction results from various bioinformatics tools for this variant were all damaging (Figure 3C). The variant amino acid is completely conserved among the six known RAB11A homologs other than zebrafish (Figure 3D).

### 3.4 Zebrafish genetic manipulation

Next, we established a zebrafish model to replicate *RAB11A* deficiency. The zebrafish genome has two *RAB11A* orthologs, rab11a (ENSDARG00000041450) and rab11al (ENSDARG00000014340). The ortholog evaluation was conducted using the DIOPT Ortholog Finder (Hu et al., 2011). The protein identity between human *RAB11A* and zebrafish ortholog genes is 95% for rab11a and 84% for rab11 al, respectively. In this study, we focused on rab11a due to its higher DIOPT score and protein identity with human *RAB11A*.

**FIGURE 4:**
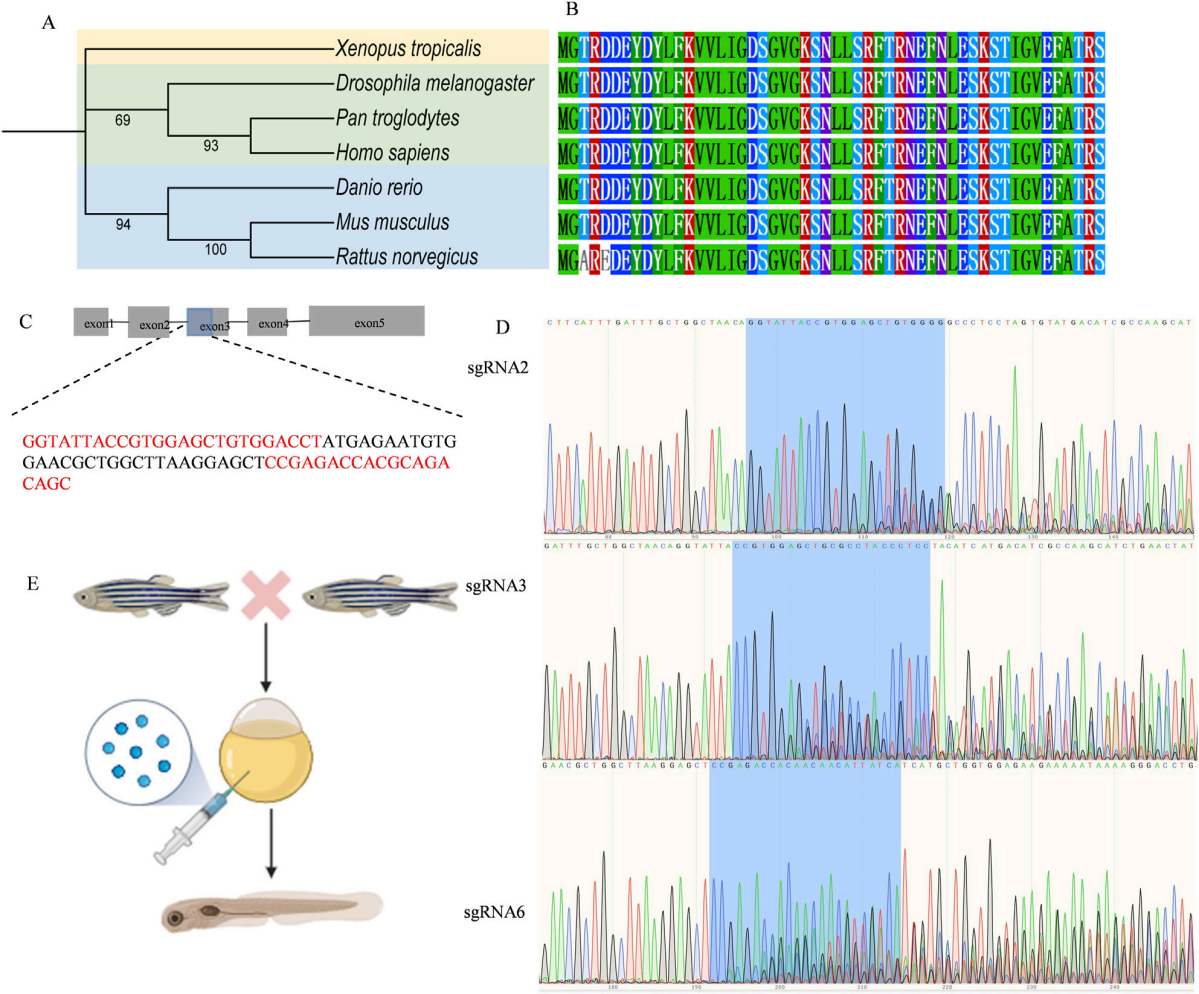
Zebrafish genetic manipulation **(A)** Phylogenetic analysis of *RAB11A* in Zebrafish, Human, and Other Species. The phylogenetic tree was constructed using amino acid sequences of *RAB11A* homologs, generated with MEGA 12 via the Neighbor-Joining method (Bootstrap = 1000 replicates) and visualized using iTOL. Branch node values indicate Bootstrap support rates (shown if ≥ 50%). Scale bar represents amino acid substitutions per site. **(B)** Amino Acid Sequence Alignment of Key Functional Domains in RAB11A Homologs Across Species **(C)** Schematic of the rab11a gene (ENSDARG00000041450) and the red sequences represent sgRNA2, 3, 6. The bule part of exon 3 represents the sgRNA knockout target site. **(D)** The Sanger sequence of mutant *rab11a* edited by sgRNA2, 3, 6. **(E)** The procedure of the establishment of rab11a deficient zebrafish model.

To resolve the evolutionary relationships of *RAB11A* in vertebrates, we analyzed homologous sequences from seven key species, including human and zebrafish (Figure 4A). The phylogenetic tree reveals that zebrafish rab11a (AAI64310.1) clusters within a distinct teleost branch (support ≥95%), forming a sister-group relationship with tetrapods (including *H. sapiens*, *M. musculus*, and *X. laevis*; node support = 98%). This indicates conserved core functionality despite ∼450 million years of divergence. Zebrafish *rab11a* serves as a robust model for studying human *RAB11A* function, with high evolutionary conservation in core functional domains. Multisequence alignment reveals complete conservation of GTPase domains (G1-G5) between humans and zebrafish, indicating similar GTP hydrolysis and switch mechanisms (Figure 4B). High conservation of zebrafish rab11a protein in critical functional domains validates its utility for modeling human RAB11A protein disorder. Single guide RNA (sgRNA) targets were identified using the CHOPCHOP online tool (Labun et al., 2019) and synthesized by GenScript. Six sgRNAs were designed for the target gene, as Supplementary Table S1. CRISPR complexes consisting of each single sgRNA (90 ng/μL) and Cas9 protein (GenScript, 250 ng/μL) were injected into fertilized embryos at the 1-2 cell stage, with a volume of approximately 1 nL. 24 h post-injection, a few embryos from the injected group were pooled and sanger sequenced to verify the mutagenesis efficacy using the TIDE (Tracking of Indels by DEcomposition) online tool (Brinkman et al., 2014) (Supplementary Table S2). sgRNAs 1,4 and 5 were excluded due to low editing efficacy (<10%), while sgRNAs 2,3 and 6 were selected for subsequent experiments. The sequence of the sgRNAs knockout target sites was shown in Figures 4C,D. To generate mutagenesis in the target gene, fertilized embryos (1-2 cell stage) were injected with approximately 1 nL of CRISPR complexes composed of three sgRNAs (120 ng/μL for each sgRNA) and cas9 protein (GenScript, 250 ng/μL) (Hwang et al., 2013) (Figure 4E). Post hoc genotyping was performed after phenotypic studies. Individual larvae were collected for sequencing and TIDE analysis to confirm the mutagenesis. And the control larvae were injected with Cas9 protein only.

### 3.5 Morphology characteristics in rab11a crispant zebrafish

The phenotypes of human *RAB11A* mutations include craniofacial malformations and developmental abnormalities. To examine the effect of *rab11a* deficiency on zebrafish morphology, morphological measurements were performed on *rab11a* deficient groups (n = 30) and Cas9 control (n = 20) (Supplementary Figure S1). There was no significant difference in eye distance between Cas9 control group and *rab11a* deficient group (p = 0.151, unpaired t-test) (Supplementary Figures S1B,S1C). Difference in body length between the two groups was also negligible (p = 0.0605, unpaired t-test) (Supplementary Figure S1D,S1E). As shown in Supplementary Figure S1F, there was a significant difference in the ratio of eye distance to body length between the Cas9 control group (n = 20, unpaired t-test) and the rab11a crispant group (n = 30) in zebrafish (p = 0.0173, unpaired t-test).

### 3.6 Brain area reduction in *rab11a* crispant zebrafish

**FIGURE 5:**
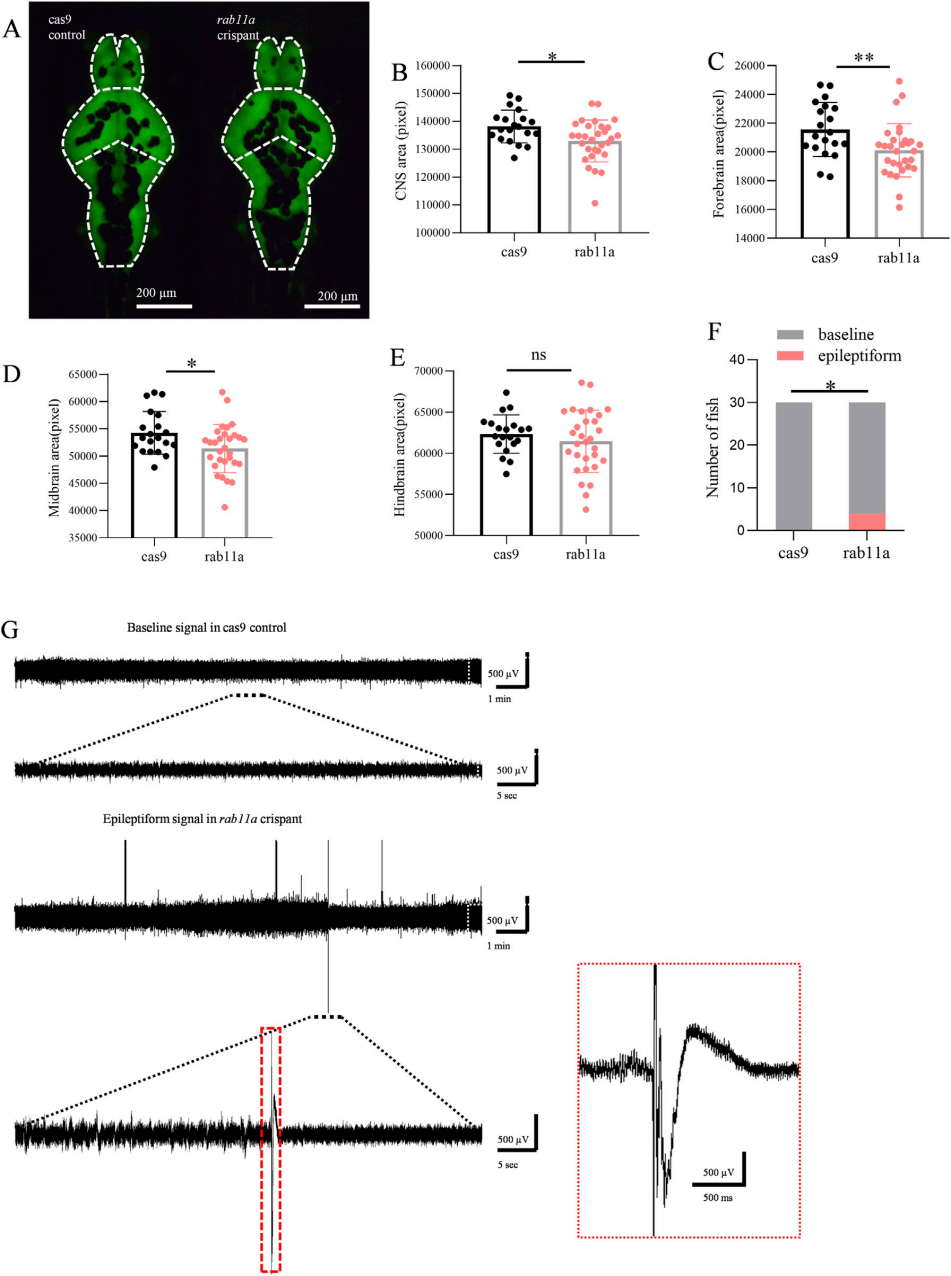
*Rab11a* deficient induces phenotypic anomalies in zebrafish larvae **(A)** Representative images of zebrafish larvae expressing HuC:eGFP. Brain fluorescence pattern at 5 dpf is displayed (dorsal view). Left, control injected with cas9; right, *rab11a* crispant. The white dashed line shows the outline of the CNS, which is divided into three parts: forebrain, midbrain, and hindbrain. **(B–D)** Measurements of brain fluorescence area in control vs *rab11a* crispant. **(B)** comparison of brain area data distribution between Cas9 control group and *rab11a* crispant group; **(C)** comparison of forebrain area data and frequency distribution between Cas9 control group and *rab11a* crispant group; **(D)** comparison of midbrain data between Cas9 control group and *rab11a* crispant group. **(E)** comparison of hindbrain data between Cas9 control group and rab11a crispant group. **(F,G)** Representative local field potential recording showing spontaneous epileptiform events in *rab11a* crispants. Data were normalized to the mean values of the control group. Error bars indicate standard deviation. Data are shown as mean ± S.E.M. *P < 0.05, **P < 0.01.

Compared to the control group injected with Cas9 (n = 20 fish), *rab11a* crispant zebrafish larvae (n = 30 fish) exhibited a significant reduction in brain area (p = 0.012, unpaired t-test). A trend toward reduced forebrain size (p = 0.009, unpaired t-test) and a significant decrease in the midbrain (p = 0.023, Student’s t-tests) was observed (Figures 5A–D). Hindbrain size, however, showed no significant difference (p = 0.359, Student’s t-test) (Figure 5E).

### 3.7 Epileptiform electrical signals in *rab11a*-deficient zebrafish

At 5 dpf, 4 out of 30 *rab11a* deficient zebrafish larvae exhibited spontaneous epileptiform electrical discharges (Figures 5F,G). A statistically significant difference was observed in comparison to the control group in which all 30 fish displayed normal baseline activity. This result suggested that the *RAB11A* mutation may have a significant impact on the neural activity of zebrafish larvae. The control normal baseline activity aligns with typical zebrafish behavior at this stage (Figure 5F), as zebrafish larvae develop different behaviors, including emotions and goal-directed behavior, by 4–5 dpf. This finding underscores the utility of the zebrafish model for studying the effects of *RAB11A* mutation on neurodevelopment and could provide valuable insights into the underlying mechanisms associated with neurodevelopmental disorders.

## 4 Discussion

The RAB11A gene is associated with many diseases, such as NDDs, neurodegenerative diseases, tumors, and inflammatory bowel disease (Joseph et al., 2023; Mignogna et al., 2023; Yoshida et al., 2024). Our study presents a case of a neurodevelopmental disorder associated with a *RAB11A* mutation featuring brain abnormalities, thinning of the corpus callosum, obesity, and abnormal muscle tone. Protein structure prediction showed that the RAB11A variation might impair the stability and activity of the RAB11A protein. Moreover, we established a *rab11a* deficient zebrafish model which recapitulates the phenotypes of patients.

The proband’s phenotype aligns with previous reports, where *RAB11A*-related NDDs were shown to affect gait, muscle tone, brain anatomy and physiology, vision, adrenarche, and body weight and structure, with some patients experiencing epilepsy (Borroto et al., 2024). For all the reported 17 children with *RAB11A* mutations, we noticed that five children with microcephaly experienced a series of OFC measurements. Four of them were diagnosed with acquired microcephaly and their brain image showed progress in brain atrophy and agenesis of the corpus callosum. Therefore, emphasis should be placed on studying the evolution of illness to achieve a comprehensive understanding of the disease. Borroto et al. reported a girl who harbors the same *RAB11A* mutation c.370A>G (Borroto et al., 2024). While we found that the phenotypes are not identical between the girl and the patient (our proband). They both had developmental disorders, brain MRI abnormalities and gastrointestinal diseases. Moreover, we found acquired microcephaly, the motor disability and obesity of the patient in the present study which was not exhibited for the girl. The detailed description of our proband with four-year follow-up would further enrich our understanding for this disease. Given the limited number of reported cases, more details of additional patients and longer follow-up are required to elucidate specific genotypes and phenotypes.

*RAB11A* mutations have been found to be associated with neurodevelopmental disorders, but animal models are lacking for validation. To address it, we generated zebrafish disease model. The reduced CNS area, and abnormal suggests that the rab11a deficient zebrafish model can well simulate *RAB11A*-related neurodevelopmental disorders. The global *Rab11a* ^null^ mouse model constructed by T. Sobajima *etal* was embryonic lethal, and the global *Rab11a* +/- mouse model has not been described in their article (Sobajima et al., 2014). Their laboratory established a *Rab11a* brain-KO (BKO) mouse model, but did not find abnormal brain development in the related model. This may be related to the fact that only the brain area of adult-BKO mice was observed in this article, and there is a lack of relevant statistical data support. No relevant behavioral studies were conducted on this *Rab11a* BKO mouse model. In addition, whether the global *Rab11a* knockout model is due to functional defects in multi-systems that cause abnormalities in the nervous system of this model is also worthy of further discussion. In the past, the global *rab11a* dominant negative zebrafish model showed abnormal embryonic curvature, but the detailed neural phenotype was not described (Clark et al., 2011). While Rab11 null mouse embryo is lethal, the rab11a and rab11ba double-ko zebrafish model generated by Hao, Y., et al. survived at 36hpf (Hao et al., 2020). It might due to that zebrafish possess four paralogs, *rab11a* (*rab11aa*), *rab11al* (*rab11ab*), *rab11ba* and *rab11bb* (Zhang et al., 2019). These collateral homologous genes may have overlapping functions (Hall et al., 2017).

Since the efficient crispant has been developed, it has broad application for the rapid identification of new genes involved in many biological processes (Shah et al., 2015; Hoshijima, K., et al., 2019; Bek et al., 2021). Recently, this approach was used for neurodevelopmental diseases more and more (Gong et al., 2022; Shu et al., 2023; Xiao et al., 2025). More genes associated neurodevelopmental diseases were known, the zebrafish crispant would offer a robust pipeline for rapidly characterizing candidate human disease genes with advanced improvement (Lin et al., 2025). To our knowledge, this is the first animal model of neurodevelopmental disorders with *RAB11A* deficiency. Future research could focus on exploring the specific functions of *RAB11A* and assessing the effectiveness of potential treatment approaches in the model, such as the relationship between autophagy, endosome–lysosome transport and neurodevelopmental and neurodegenerative diseases (Sultana and Novotny, 2022). In this study, we only validated the neurologic phenotype in a zebrafish model, the motor disorder, obesity, behavior problems and other multisystemic phenotypes may be further investigated. However, it should be noted that we did not establish a rab11a N124D zebrafish model. We investigated the prominent phenotypes of reduced brain size and hyperexcitability. Additional behavioral tests, such as novel object recognition and three-chamber social interaction tests, will be conducted when feasible.

In summary, we present a case of a *RAB11A*-related NDD patient, predominantly characterized by global developmental delay, motor disorder and brain anomalies. Additionally, we have generated a *rab11a* deficient zebrafish model to study neurodevelopmental disorders associated with RAB11A gene mutations.

## Data Availability

The WGS datasets presented in this article are not readily available because privacy-related concerns with sharing the data. Requests to access the datasets should be directed to the corresponding author through email.
