# Supplementary material for: A de novo mutation in RAB11A is associated with neurodevelopmental disorder accompanied by variable multisystem abnormalities
Source: Front Genet. 2025 Sep 1;16:1636206. doi: 10.3389/fgene.2025.1636206 (PMC12434753; doi:10.3389/fgene.2025.1636206)
Supplement: Supplementary file 1 [file DataSheet1.docx]

| **Supplemental data** | |
| --- | --- |
| Supplementary Figure 1 | Morphology characteristics of zebrafish |
| Supplementary Table 1 | Oligonucleotides |
| Supplementary Table 2 | SgRNA editing efficiency by TIDE |
| Datasets | https://pan.quark.cn/s/255881942b79 |


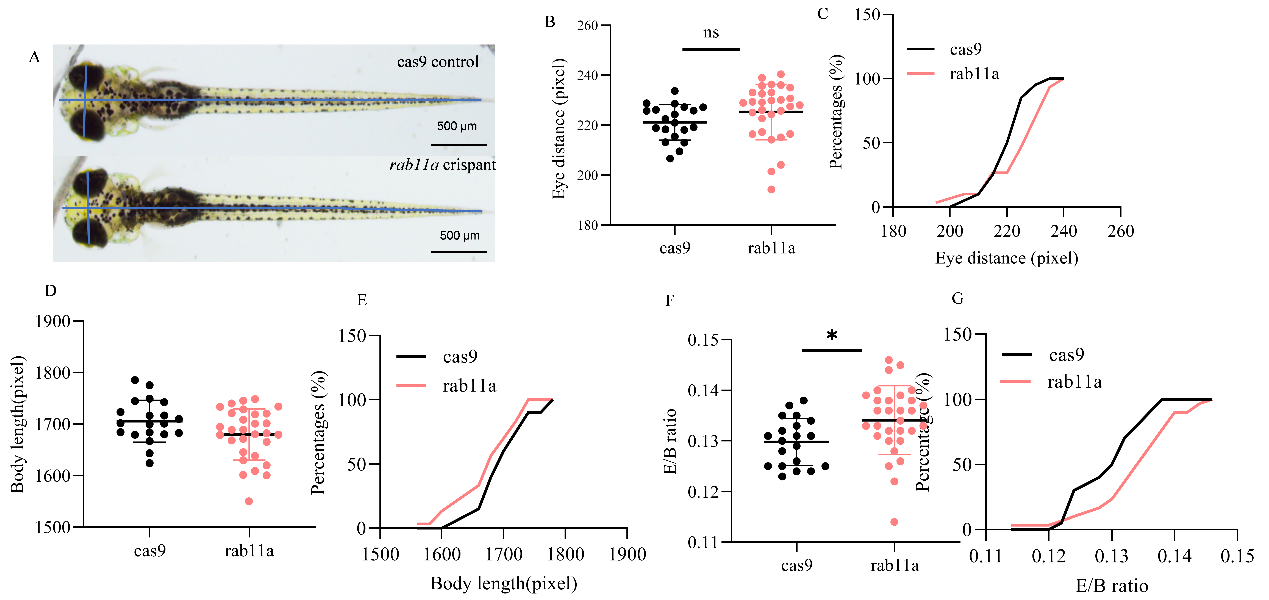


**Supplementary Figure 1. Morphology of zebrafish**

(A) Light field photos of Cas9 control group and *rab11a* deficient group zebrafish. (B-C) comparison and frequency distribution of eye distance data between Cas9 control group and *rab11a* crispant group. (D-E) comparison and frequency distribution of body length data between Cas9 control group and *rab11a* crispant group. (F-G) Comparison of eye distance/body length ratio data and frequency distribution between Cas9 control group and *rab11a* crispant group. *P < 0.05.

**Supplementary Table 1 Oligonucleotides**

| Name | Sequence |
| --- | --- |
| sgRNA1 | CTGGCTAACAGGTATTACCGTGG |
| sgRNA2 | GGTATTACCGTGGAGCTGTGGGG |
| sgRNA3 | GGAGGGCCCCCACAGCTCCACGG |
| sgRNA4 | GCTTGGCGATGTCATACACTAGG |
| sgRNA5 | GAATGTGGAACGCTGGCTTAAGG |
| sgRNA6 | TGTTGCTGTCTGCGTGGTCTCGG |
| rab11a-F1 | GCTTAACCCTAGGAATACTGGTGA |
| rab11a-R1 | ACTCACCAGTCAGGATGGTCT |
| rab11a-F2 | TTAACCCTAGGAATACTGGTGAGGC |
| rab11a-R2 | TCTCTAGGAAGGACAGACCGT |

**Supplementary Table 2** **SgRNA editing efficiency by TIDE**

| ID | sgRNA1 | sgRNA2 | sgRNA3 | sgRNA4 | sgRNA5 | sgRNA6 |
| --- | --- | --- | --- | --- | --- | --- |
| Sample 1 | 0% | 18% | 34% | 3% | 7% | 50% |
| Sample 2 | 2% | 9% | 12% | 18% | 7% | 38% |
| Sample 3 | 0% | 13% | 9% | 0% | 4% | 48% |
| Average | 1% | 13% | 18% | 7% | 6% | 45% |
